# Release of Soybean Isoflavones by Using a β‐Glucosidase from *Alicyclobacillus herbarius*

**DOI:** 10.1002/cbic.202000688

**Published:** 2020-12-30

**Authors:** Lidia Delgado, Christian M. Heckmann, Flavio Di Pisa, Louise Gourlay, Francesca Paradisi

**Affiliations:** ^1^ University of Nottingham, School of Chemistry Department of Chemical Biology University Park Nottingham NG7 2RD UK; ^2^ Dipartimento di Bioscienze Università di Milano Via Celoria 26 20133 Milan Italy; ^3^ University of Bern Department of Chemistry and Biochemistry Freiestrasse 3 3012 Bern Switzerland

**Keywords:** biocatalysis, extremophiles, hydrolases, isoflavones, soy

## Abstract

β‐Glucosidases are used in the food industry to hydrolyse glycosidic bonds in complex sugars, with enzymes sourced from extremophiles better able to tolerate the process conditions. In this work, a novel β‐glycosidase from the acidophilic organism *Alicyclobacillus herbarius* was cloned and heterologously expressed in *Escherichia coli* BL21(DE3). *Ahe*GH1 was stable over a broad range of pH values (5–11) and temperatures (4–55 °C). The enzyme exhibited excellent tolerance to fructose and good tolerance to glucose, retaining 65 % activity in the presence of 10 % (*w/v*) glucose. It also tolerated organic solvents, some of which appeared to have a stimulating effect, in particular ethanol with a 1.7‐fold increase in activity at 10 % (*v/v*). The enzyme was then applied for the cleavage of isoflavone from isoflavone glucosides in an ethanolic extract of soy flour, to produce soy isoflavones, which constitute a valuable food supplement, full conversion was achieved within 15 min at 30 °C.

## Graphical abstract

**Tough guy**: Enzymatic characterisation showed that a β‐glycosidase from *Alicyclobacillus herbarius* was stable over a broad range of pH values and temperatures, also exhibiting excellent tolerance to sugars and organic solvents, specially to ethanol. The enzyme was applied in the hydrolysis of isoflavone glucosides in an ethanolic extract of soy flour with outstanding results.

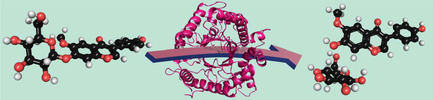

## Introduction

β‐Glucosidases (EC 3.2.1.21) constitute a group of enzymes that hydrolyse terminal, non‐reducing glycosyl residues from glycosides.^[1]^ These enzymes have been successfully applied in a broad range of industrial applications,^[2]^ and they have become key tools to hydrolyse very stable glycosidic bonds in a clean and efficient way.

However, many food industrial processes involve harsh conditions (high concentrations of solvents and sugars, low pH and high temperatures) that can inactivate enzymes.[^3^, ^4^] Extremophiles are organisms well adapted to extreme environmental conditions^[5]^ and they constitute a novel and alternative source of enzymes (extremozymes) for industrial applications. Extremozymes are generally more resistant in demanding industrial processes when compared to mesophilic enzymes.^[6]^

**Figure 1:**
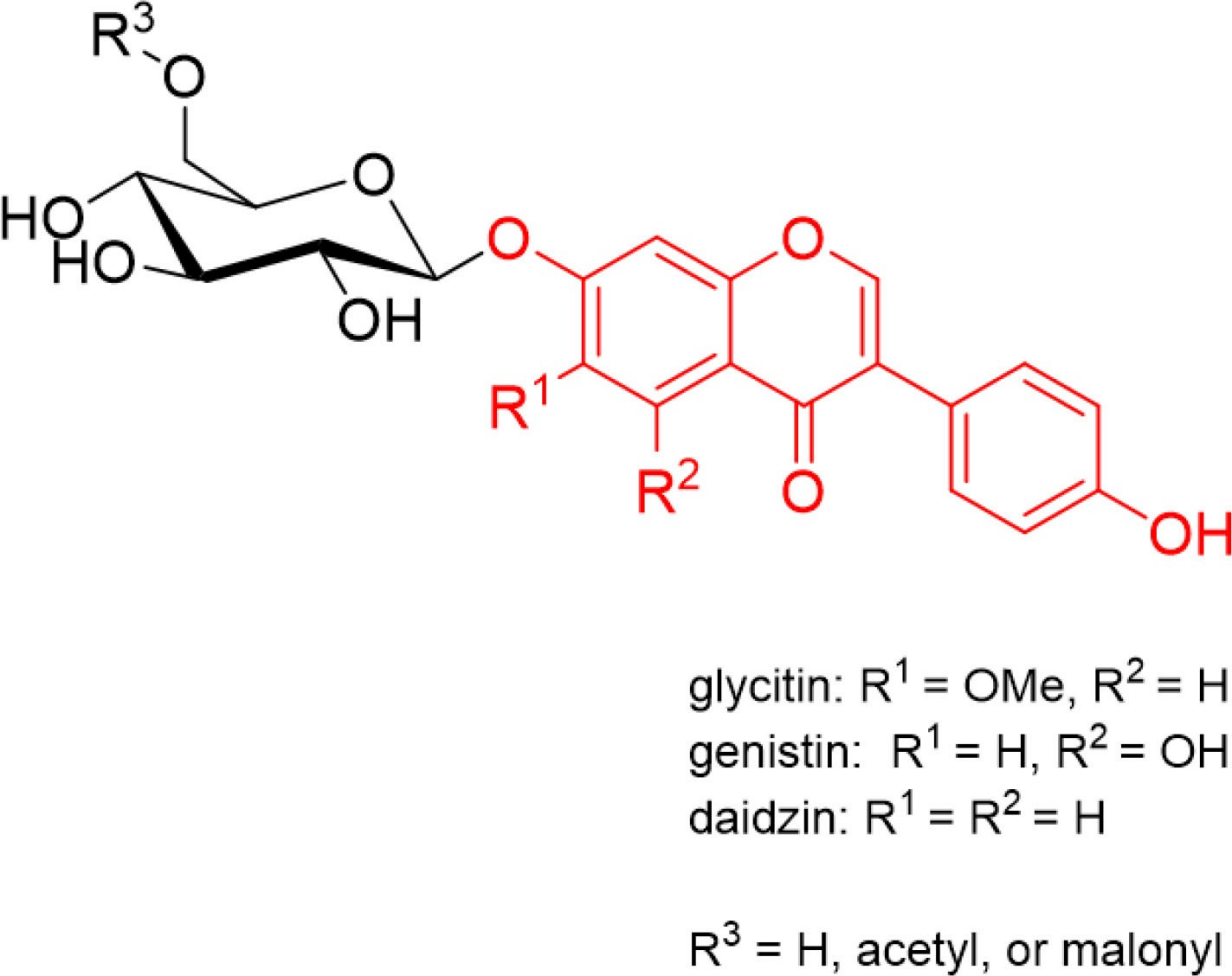
Structure of the main isoflavone glucosides (glycitin, genistin, and daidzin) found in soybean. The isoflavone moiety (glycitein, genistein, and daidzein) is highlighted in red. The sugar may be further acetylated or malonylated.

Soybean (*Glycine max*) originating in China, constitutes one of the largest sources of vegetable oil in the world and has the highest protein content among all others food crops.^[7]^ Its consumption has become increasingly popular in recent years as it is an excellent protein source for the human diet. In addition, soybean contains several compounds considered important food supplements due to their health properties, especially isoflavones. Soybeans mainly contain three types of isoflavones (daidzein, genistein, and glycitein), which can be found in four different forms (Figure 1): as aglycons, 7‐*O*‐β‐d‐glucosides, 7‐*O*‐(6′′‐*O*‐acetyl)glucosides, or 7‐*O*‐(6′′‐*O*‐malonyl)glucosides.^[8]^ Recently, commercial preparations of isoflavones have come to the public attention following studies on their reported positive effects on cognitive function.^[9]^ However, when the biological activities of these compounds are considered, the bioavailability of the aglycone has been suggested to be higher than that of the glycoside; but it represents only a minor constituent of unfermented soy products.^[10]^

β‐Glucosidases can be used to hydrolyse isoflavone glucosides to their aglycons. The industrial processing for extracting isoflavones from soybeans includes the use of organic solvents (mostly ethanol) which is necessary to solubilise the isoflavones.^[11]^ Soy isoflavones are released as a side‐product during the industrial fermentation process for the production of erythromycin, using the microorganism *Saccharopolyspora erythraea* (by endogenous β‐glucosidases). However, the isoflavone aglycones themselves are metabolised by that organism, requiring either metabolic engineering or subsequent enzymatic treatment (or acid hydrolysis) of the spent broth to obtain the aglycon.[^12^, ^13^, ^14^] The use of purified β‐glucosidases for isoflavone aglycon production from soy flour has also been investigated, in particular from the thermophilic bacteria *Thermotoga maritima* and *Thermoanaerobacter ethanolicus* JW200. However, these enzymes required defatting of the soy flour using hexane (an undesirable neurotoxic solvent^[15]^) and high reaction temperatures (65–80 °C).[^16^, ^17^] The use of a GH1 β‐glucosidase from *Alicyclobacillus* sp. A4 as a supplement in soy‐based animal feed to aid in the release of the aglycon in monogastric animals has also been investigated under milder conditions (37 °C), but in the absence of the co‐solvents required for extracting the aglycons.^[18]^ Again, many β‐glucosidases reported to date from mesophilic organisms are inhibited by both organic solvents.^[19]^ and glucose,[^20^, ^21^] which would be present at increasing concentrations as the hydrolytic process progresses.

In this work, a novel β‐glucosidase (WP_026963033.1) of the glycosyl hydrolase family 1 (GH1) from the extremophilic organism *Alicyclobacillus herbarius* (*Ahe*) has been identified and investigated. *Ahe* was first isolated from a herbal tea made from dried flowers of hibiscus, it has been described as a thermo‐acidophilic Gram‐positive bacterium that grows at a range of temperatures between 35–65 °C and a pH between 3.5 and 6, features that are very appealing for different applications in the food industry market.^[22]^ The enzyme has been cloned and expressed in *Escherichia coli*, its crystal structure has been solved and the enzyme has been characterised to assess its performance under different operational conditions (glucose, fructose, organic co‐solvents and range of pH values and temperatures) usually found in food industrial processes. Following the initial characterisation, *Ahe*GH1 has been applied to hydrolyse the main isoflavone glucosides present in soybean flour.

## Results and Discussion

### Protein expression and purification

*Ahe*GH1 was expressed and purified with excellent yields between 75–100 mg/L of culture. The purification, by metal affinity chromatography, was analysed by SDS‐PAGE (Figure S1 in the Supporting Information). *Ahe*GH1 has a theoretical monomeric molecular weight of 52 133.66 Da (≈52 kDa) estimated by the online tool ProtParam^[23]^ and sufficient degree of purification was confirmed by SDS‐PAGE. The molecular weight was determined to be 99.7 KDa by gel filtration, consistent with it being a dimer in solution (Figure S2 and Table S1).

### The 3D structure of AheGH1

**Figure 2:**
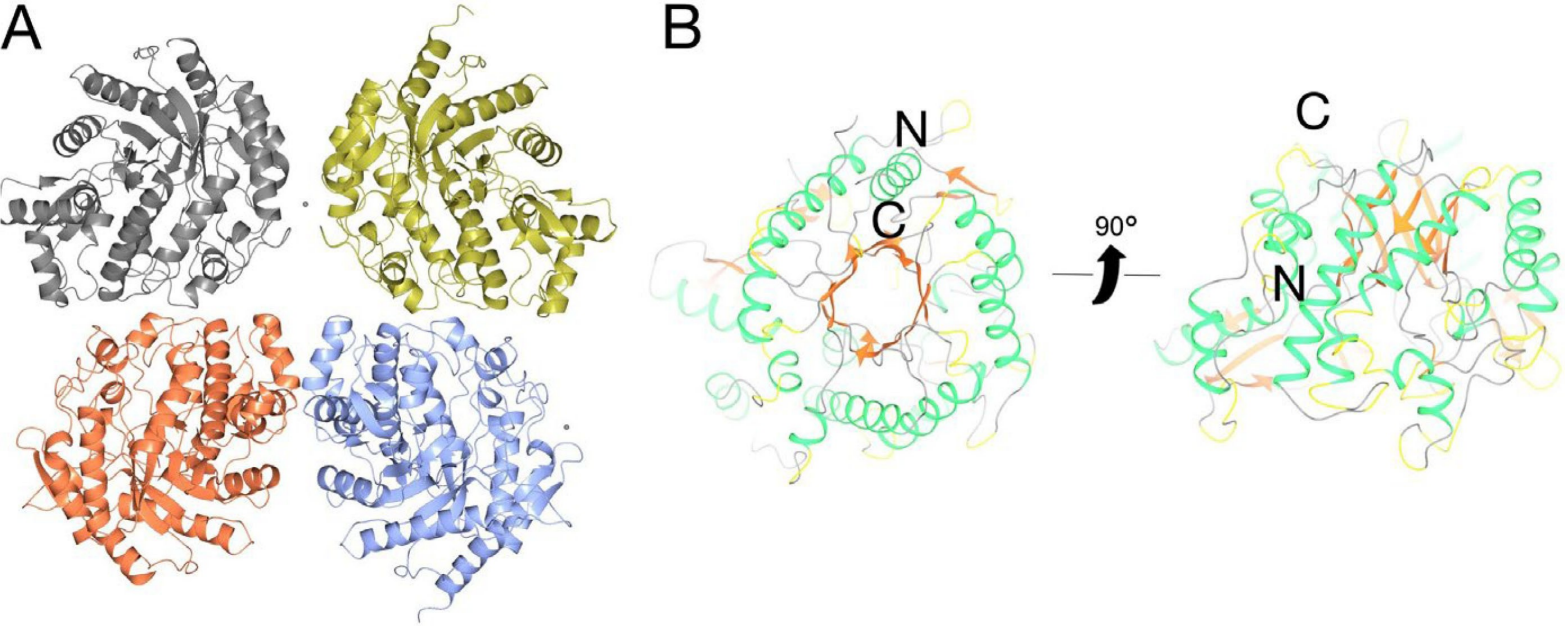
3D structure of *Ahe*GH1. A) The *Ahe*GH1 tetramer present in the asymmetric unit. B) Top and side views of the *Ahe*GH1 monomer, with ribbons coloured according to secondary structure (β‐strands in orange, α‐helices in green, 3‐, 4‐ and 5‐turns in yellow, unstructured regions in grey). N‐ and C‐terminal regions are labelled.

Crystals of *Ahe*GH1 enzyme belonging to the monoclinic space group P21 diffracted at 1.98 Å resolution. The asymmetric unit of *Ahe*GH1 contains four independent molecules, with a calculated Matthews coefficient of 2.42 Å^3^ Da^1^ (estimated solvent content of 49.31 %; Figure 2). The overall structure of the four *Ahe*GH1 subunits is identical (RMSD values of 0.05 to 0.08 Å over 356 backbone Cα atoms).

The final model was refined to *R* values of *R*_work_ 24.7 % and *R*_free_ 29.8 %. The presence of strong translational noncrystallographic symmetry (tNCS), as confirmed by a peak in the Patterson map, may justify the higher than expected final *R*_work_ value, despite the high resolution of the structure (Table S2). Electron density map was overall of good quality, with electron density coverage across residues 2‐450 (chain A), 2‐451 (chain B), 3‐447, (chain C) and 3‐446 (chain D), except for a short stretch (P304‐D322). Several ethylene glycol molecules derived from the crystallisation buffer were modelled into the electron density. In addition, two nickel cations were identified in the model, present during the affinity chromatography purification step. The metal cations are located at the dimer interfaces between chain A and the symmetry‐related monomer C and between the B and D subunits and are coordinated in a tetrahedral arrangement by the side chains contributed from two histidine (H61) and two glutamate (E29) residues.

Ahe*GH1* is arranged into a single (β/α)_8_ barrel fold, common to this family of glycosidases, with negligible main‐chain displacements in peripheral loops and α‐helices. Regions which show more significative changes in the secondary structure correspond to residues 272 to 281, folded into a gamma and two β‐turns, and residues 409 to 416 that form two β‐turns instead of the more common α‐helices (Figure 2).

A feature of the GH1 family is that despite low sequence identity (as low as 17 %) they share high structure conservation. 3D structure‐based comparisons performed with the DALI server (http://ekhidna2.biocenter.helsinki.fi/dali/)^[24]^ revealed that the *Ahe*GH1 protein has the highest structural similarity with a family 1 glucoside hydrolase from *Paenibacillus polymyxa* (*BglB*; PDB ID: 2O9P).^[25]^ Despite average sequence identity (52 %), superposition between the two proteins revealed a high degree of structural similarity (RMSD of 0.6 Å) over 441 aligned residues.

### The AheGH1 active site

**Figure 3:**
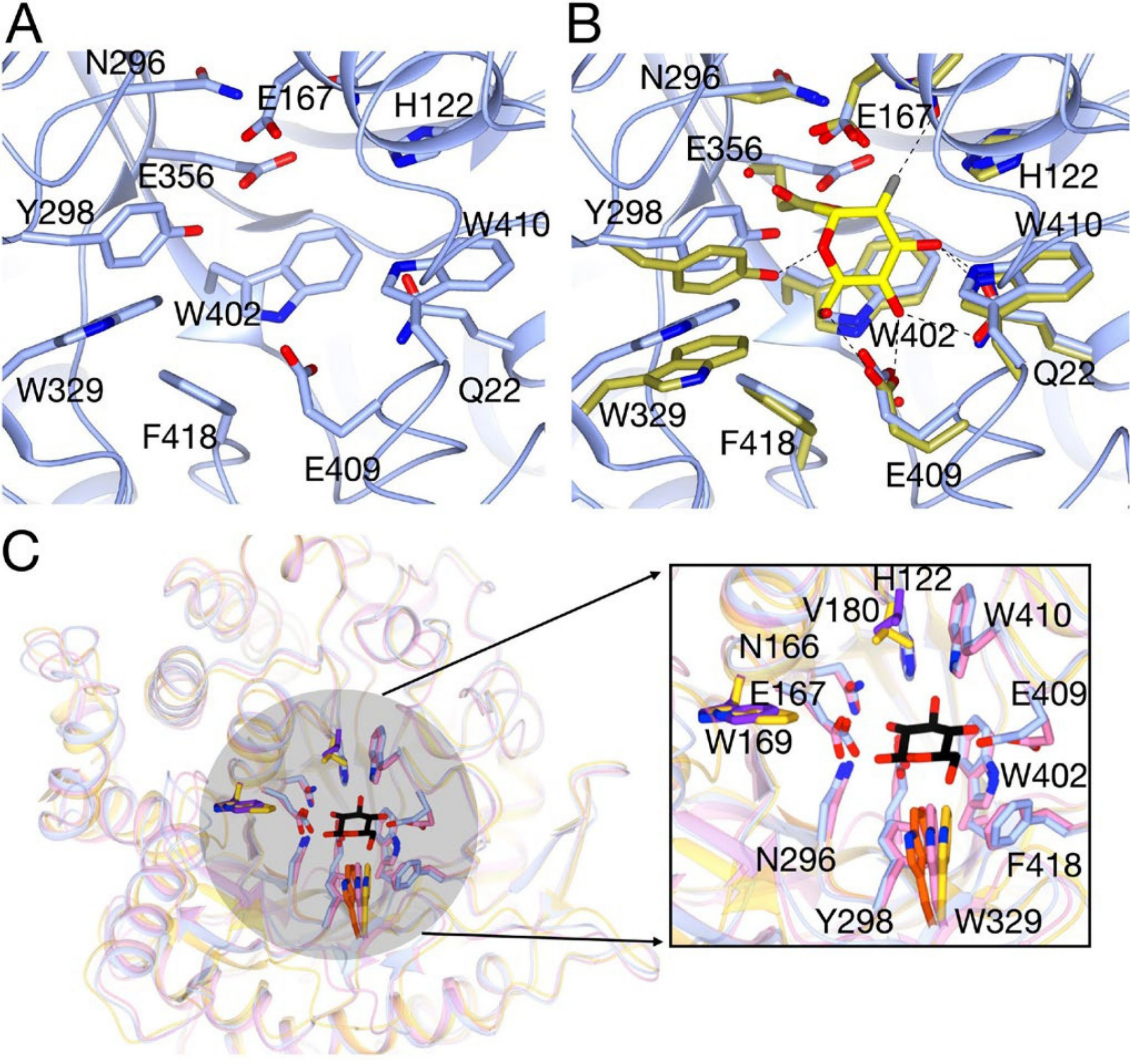
The *Ahe*GH1 active site. A) The *Ahe*GH1 putative active site showing active‐site residues delineating the pocket (sticks). Proposed catalytic resides E356 and E167 are indicated. B) Structural superposition of the active sites of *Ahe*GH1 (blue) and the homologous protein *BglA* (gold; PDB ID: 1E4I) complexed with 2‐deoxy‐2‐fluoro‐α‐d‐glucopyranose catalytic intermediate (yellow sticks) and C) superposition of *Ahe*GH1 (ice blue) with *BglA* (yellow) and *BglB* (pink) in complex with glucose (PDB ID: 2O9T) and a detailed view of the glucose binding site. Glucose is shown as black sticks. *BglB* residues interacting with the glucose molecule, and the corresponding *Ahe*GH1 residues, are depicted as sticks and coloured accordingly. *Ahe*GH1 residues conserved in *BglA* are coloured in purple. *Ahe*GH1 W329, which is conserved in all the three homologues is coloured in orange. Residue numbering in all panels is for *Ahe*GH1. This figure was made with CCP4mg.^[30]^

An enzyme template search of catalytic site templates made with the ProFunc server (http://www.ebi.ac.uk/thornton‐srv/databases/ProFunc),^[26]^ identified the cyanogenic family 1 glycosyl hydrolase (CBG) from white clover (PDB ID: 1CBG)^[27]^ as the top hit. *Ahe*GH1 and CBG share low (36.94 %) overall sequence identity, yet high (73.47 %) local sequence identity in their active sites over 49 equivalenced residues. At a structural level, they share high structural similarity (99.5 % over 493 matched residues) and as for *Ahe*GH1, CBG is a homodimer in solution.^[27]^ In agreement with CBG and other GH1 members in general, the active site pocket contains several conserved polar and aromatic residues that are typically present in carbohydrate recognition sites that binds the nonreducing end of the substrate.^[21]^ Based on comparisons with GH1 members, *Ahe*GH1 the main active site residues present are: R79, H122, E167, N166, N296, Y298, E356 and W402 (Figures 3 and S3+4).

The mechanism of catalysis generally described for this class of enzyme involves a double displacement reaction, requiring a proton donor and a nucleophile.^[27]^ Previous results are consistent with an ionised carboxylic acid group acting as a catalytic nucleophile and a histidine residue or carboxylic acid group (with a significantly elevated p*K*_a_) behaving as a general acid catalyst. Based on comparisons made with GH1 members in general, E167 and E356 are likely to be the acid‐base catalyst and nucleophile, respectively in the reaction (Figures 3 and S4).

With regards to the active site architecture of GH1 enzymes in general, structural and thus functional differences are attributed to the loop regions that are present between the β/α motifs that shape the active site cavity. This may be appreciated by comparison of the overall sequence and structure conservation of *Ahe*GH1 with all other enzymes of similar structure, using the ENDscript 2 server (http://endscript.ibcp.fr/ESPript/ENDscript/).^[28]^ As expected, structure and sequence divergence occurs mainly in the loop regions connecting the β/α motifs (Figure S3).

In *BlgB*, nine active‐site residues are reported to delineate the entrance to the active site: Y169, T178, E180, R243, E225, Q316, H318, W328 and W412 (*BlgB* numeration).^[25]^ These residues render the active site cavity narrower in comparison with its homologue *BglA* (PDB ID: 1E4Y).^[29]^ Except for W328, all remaining residues in *Ahe*GH1 are not conserved (Figure S4). Higher similarity, in terms of sequence identity, was on the other hand detected between *Ahe*GH1 and the *BglA* active site, defined by residues W168, L177, V179, S224, T242, E314, N316, W326 and E408. Three out of the nine residues (i. e., W168, V179 and W326; Figure S4) are conserved between the two proteins. In addition, according to the nature and steric hindrance of the side‐chains of the residues defining the active site, the *Ahe*GH1 cavity is more similar to that of *BglA*.

### Activity assay and kinetic parameters

**Figure 4:**
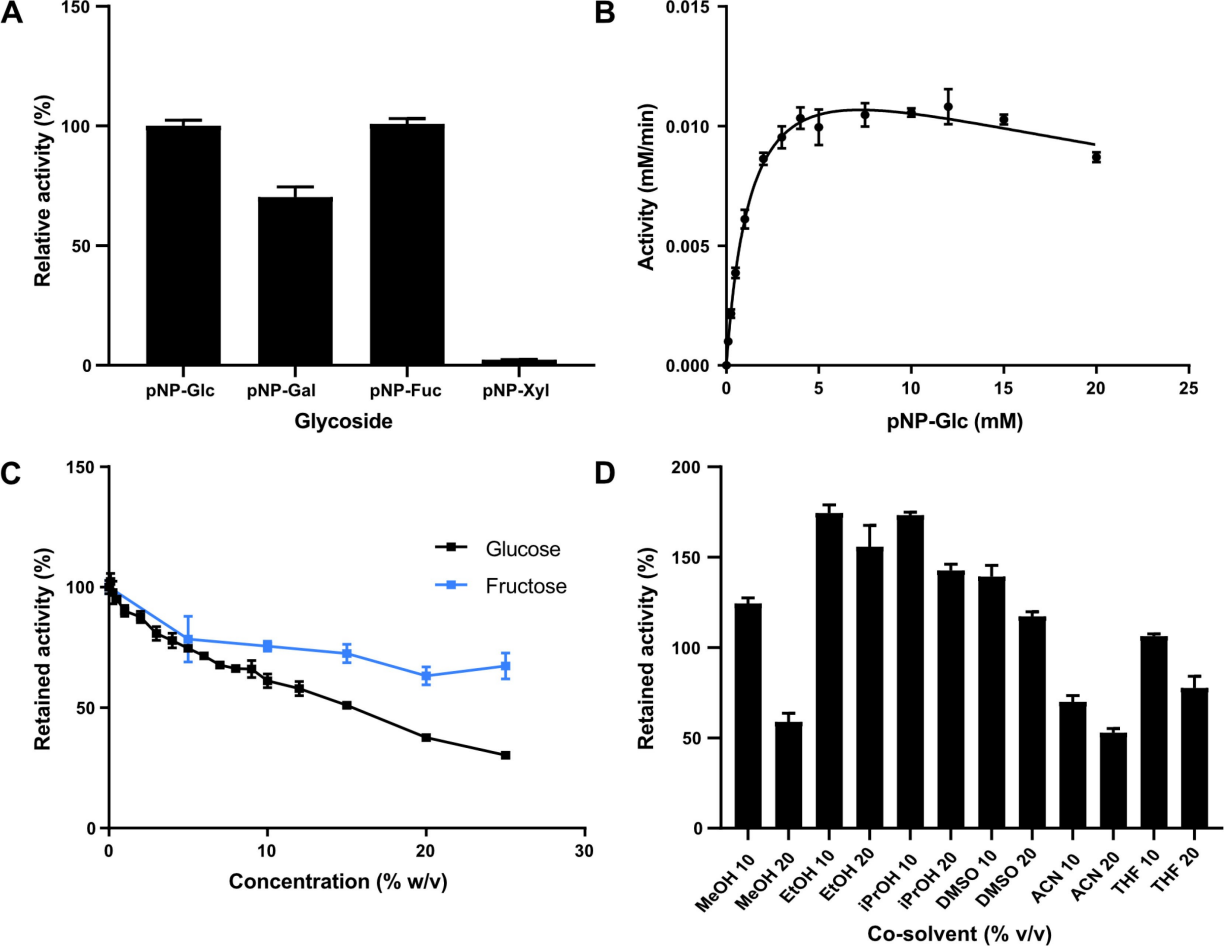
A) Specific activity with various pNP‐glucosides. B) Substrate inhibition kinetics with *p*NPG. Effects of C) glucose and fructose and D) co‐solvents on activity. Represented in the figures are the mean and the standard deviation. The activity was tested in a standard activity assay at 25 °C. Error bars represent standard deviations (*n*=3).

The activity assay was performed spectrophotometrically using *p*‐nitrophenyl‐β‐d‐glucopyranoside (*p*NPG) at 25 °C. 10 μL of the suitable dilution of the enzyme was added to a 96‐well plate in triplicate together with 0.29 mL of the reaction solution *p*NPG (10 mM), HEPES (50 mM), pH 7.4. The *p*‐nitrophenol formation was followed for 10 minutes at 420 nm. The enzyme showed a specific activity of 20 U mg^−1^. *Ahe*GH1 also showed activity with *p*‐nitrophenyl‐β‐d‐fucopyranoside, ‐galactopyranoside, and ‐xylopyranoside. While the activity with the fucoside was unchanged, it was reduced by approximately 30 % for the galactoside and 98 % for the xyloside (Figure 4A).

The kinetic parameters of *Ahe*GH1 for *p*NPG were determined under the same conditions of the activity assay but using different substrate concentrations.^[31]^ *K*_m_ (1.4±0.1 mM), *K*_i_ (37±6 mM) and *k*_cat_ (2.7±0.1 s^−1^) were calculated in triplicate by nonlinear regression using GraphPad Prism 8 (Figure 4B; see the Experimental Section for details).

### Enzymatic characterisation

A thorough characterisation of this novel enzyme was performed, to probe the activity and stability in the presence of sugars, solvents, different pH values and different temperatures.

### Activity assays

The effect of glucose, fructose and a different range of co‐solvents is reported in Figure 4.

Glucose inhibition (Figure 4C) is a common problem among β‐glucosidases,[^21^, ^32^] as the accumulation of the product of the hydrolytic process naturally reduces the catalytic efficiency of the enzyme. However, it has been reported that some β‐glucosidases belonging to the family GH1 can be exceptionally tolerant or even stimulated by glucose, but the mechanism of that tolerance/stimulation is still not clear.[^3^, ^18^, ^33^] The structure of *BglB* in complex with glucose (PDB ID: 2O9T)^[25]^ has been solved; the residues involved in ligand binding (Q22, H122, N166, E157, H181, E365, W410, E409, W412:*BglB* numbering) are conserved in *Ahe*GH1 (Figure 3C). A mechanism of product inhibition, whereby the glucose molecule remains tightly bound to the active site until it is displaced by the arrival of a new substrate molecule, has been suggested for a plant β‐d‐glucan glucohydrolase GH3 enzyme.^[34]^ Further studies to assess the behaviour of *Ahe*GH1 in the presence of glucose were carried out. A broad range of glucose concentrations, from 0.1 to 25 % (*w/v*), were evaluated, showing that the *Ahe*GH1 activity slowly decreases as the concentration of glucose increases. With 25 % (*w/v*) of glucose in the reaction, *Ahe*GH1 retains 30 % of its activity. In contrast, the β‐glucosidase from *Aspergillus niger*, a glycosidase commonly used in food industrial processes, only retains 2 % activity in the presence of 10 % (*w/v*) glucose in the mixture.^[35]^ Fructose (Figure 4C) has a milder effect on the enzyme activity than glucose. The retained activity varies between 80 %, with 5 % (*w/v*) of fructose, and 67 %, with 25 % of fructose in the reaction mixture. It is unclear whether the effect of fructose is due to inhibition or caused by the two‐ to threefold increase in viscosity as concentration increases.^[36]^

Commonly, many enzymes from mesophilic organisms are significantly destabilised by organic solvents. This could be attributed to the loss of crucial water molecules that maintain the protein conformation (desolvation), affecting the *K*_m_ and *v*_max_ values, and, in the most dramatic cases the overall protein folding. Retained activity in the presence of organic solvents is possible only when the protein surface and the active site remain well hydrated. *Ahe*GH1 presents an impressive co‐solvent tolerance, retaining over 50 % activity in all cases (Figure 4D). Particularly relevant for the intended application of this enzyme, is its performance in the presence of ethanol which is one of the most common solvents in food processing; with 10 and 20 % of ethanol in the reaction mix, *Ahe*GH1 is 50 % more active than without ethanol at all. This behaviour has been previously reported for other glucosidases in literature.^[37]^

### Stability assays

Enzyme long term stability is also a key parameter to assess the potential implementation of the catalyst in biotechnological processes. Results for the *Ahe*GH1 stability assay in the presence of co‐solvents and a different range of temperatures and pH values have been evaluated.

**Figure 5:**
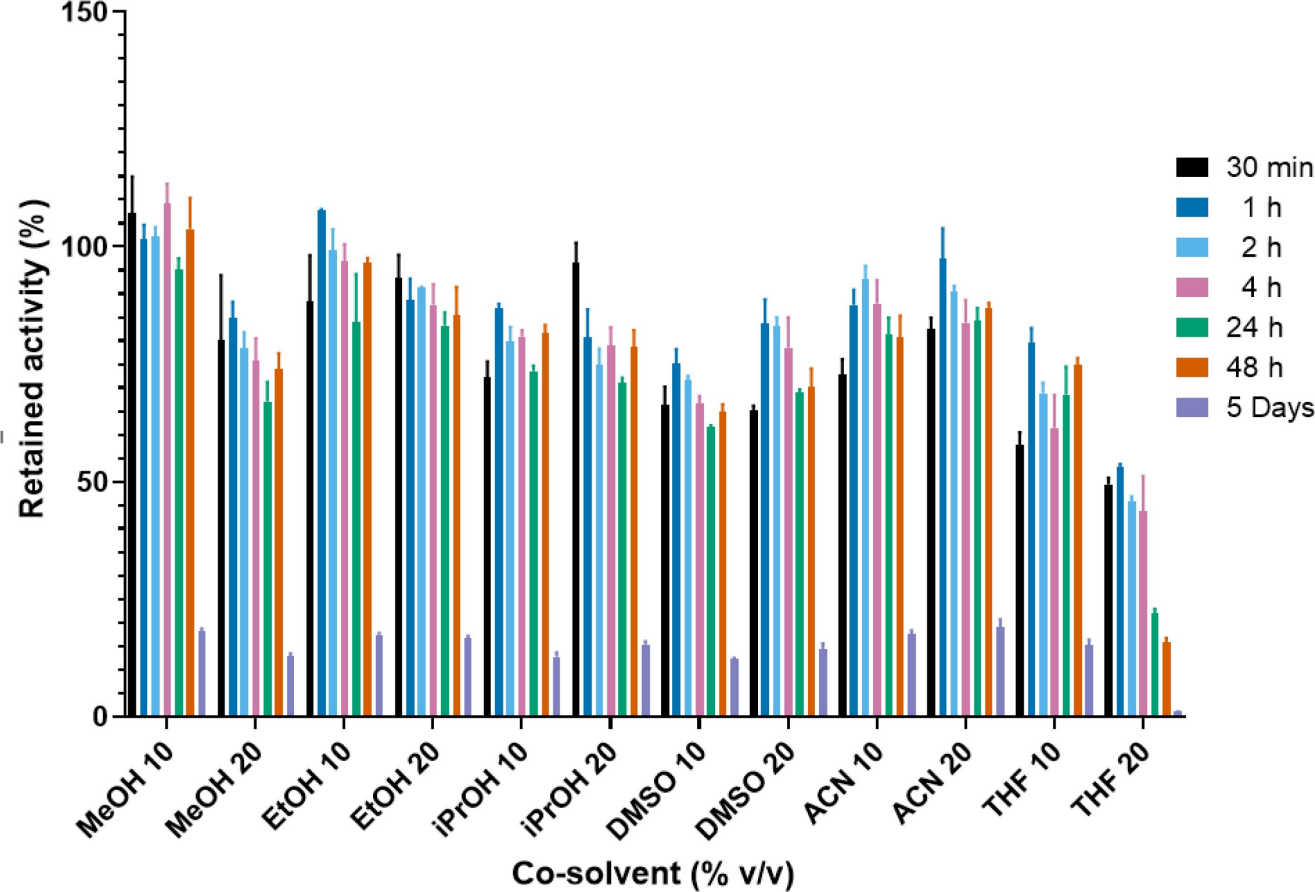
Effect of incubation in different co‐solvents on *Ahe*GH1 stability. The activity was tested in a standard activity assay at 25 °C. Activity expressed relative to the activity at *t*=0. Error bars represent standard deviations (*n*=3).

When the enzyme is incubated with co‐solvents (Figure 5), a very stable behaviour over significant period of time is observed. *Ahe*GH1 retains over 55 % activity after 48 h incubation in all 12 conditions.

The results obtained for the enzymatic stability at a different pH values (Figure S5) showed that *Ahe*GH1 is stable between pH 5 and 11, less active at pH 4 and 12 and completely unstable at pH 3. The enzyme shows no activity at pH 3 despite being selected from an acidophilic organism. Indeed, the ability of the source microorganism in dealing with acidic pH does not always translate to the isolated catalyst. Most adaptation mechanisms developed by acidophiles to survive at low pH involve very efficient homeostasis which prevents the ingress of protons to the cytoplasm.^[38]^ Consequently, the cytoplasmatic enzymes of those organism do not necessarily deal with acidic conditions and hence are not adapted to it.

Regarding stability at different temperatures (Figure S6), *Ahe*GH1 retains above 40 % activity from 4 up to 55 °C, covering the usual range of temperatures of food industrial processes. This is lower than for the GH1 from thermoacidophile *Alicyclobacillus acidocaldarius* (WP_008336965.1; 52 % identity),^[39]^ which was stable at up to 65 °C. On the other hand, the aforementioned *BglB* of *P. polymyxa* has a half‐life of 2.7 min at 55 °C. Arrizubieta and Polaina^[40]^ reported four mutations (three residues) that enhance its thermostability, H62R, M319I/V and M361I, with H62 M resulting in the largest increase in thermostability. The equivalent residues in *Ahe*GH1 are R62, A319 and F361 (see the Supporting Information). Thus, the most significant mutation in *BglB* is mirrored and the other two residues are altered, which might contribute to the enhanced thermostability of *Ahe*GH1. Yet, while the authors propose a salt‐bridge between R62 and E429 in *BglB*, the equivalent residue in *Ahe*GH1 is Q429.

The thermal stability of *Ahe*GH1 was measured in thermofluorimetry studies, following the increase in fluorescence intensity (*λ*_ex_=470–505 nm; *λ*_em_ =540–700 nm) that arises due to the binding of the fluorophore SYPRO™Orange to internally located hydrophobic residues of the protein, that become exposed during thermal denaturation (see the Experimental Section). *Ahe*GH1 unfolded in a single step, with a melting temperature (*T*_m_) of 67 °C (Figure S7). This is approximately 10 °C higher than what has been reported for wild‐type *BglB*,[^41^, ^42^] further confirming the increased thermostability of *Ahe*GH1.

### Activity of AheGH1 with isoflavone glucosides

**Figure 6:**
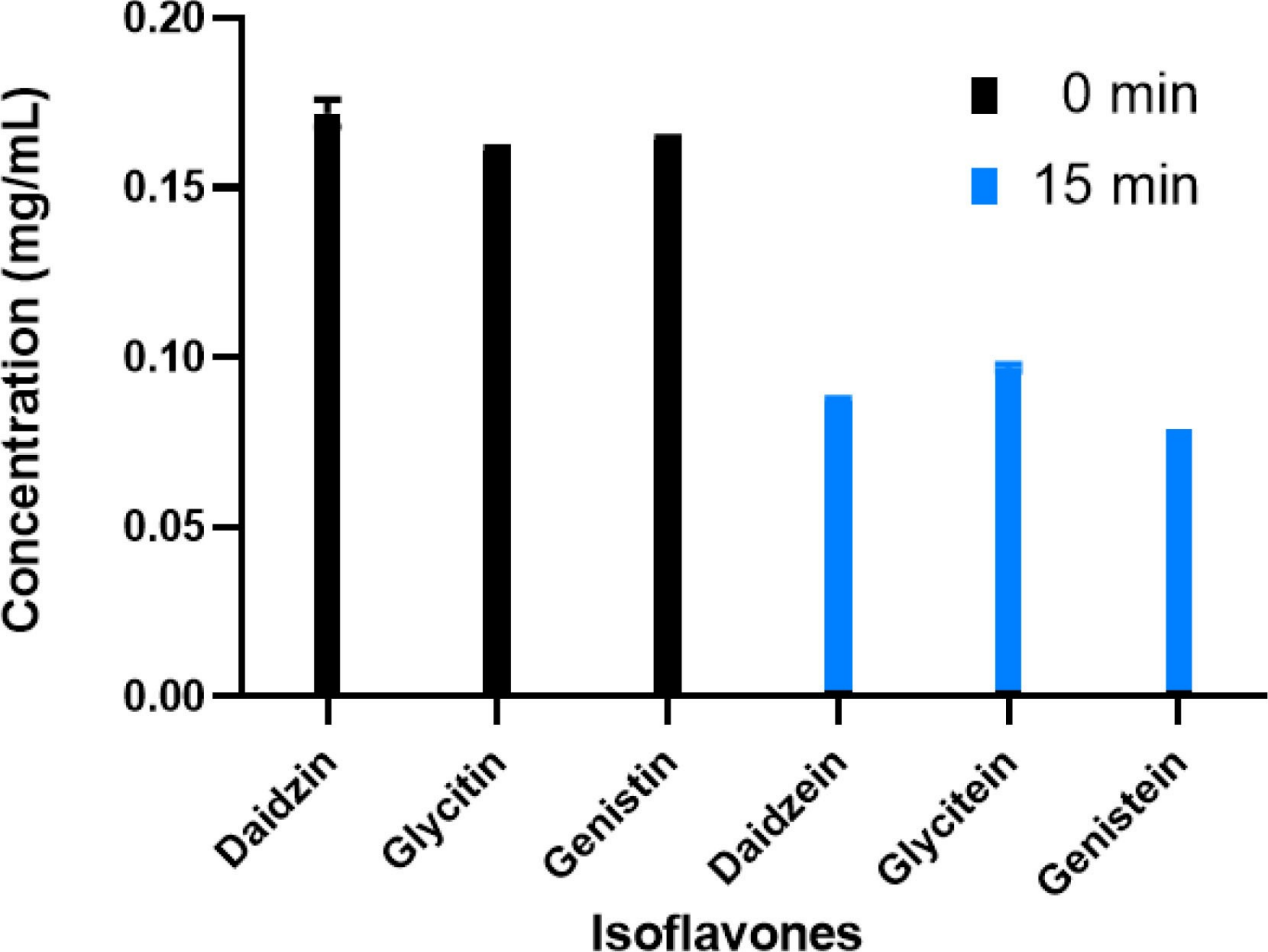
Enzymatic hydrolysis of 3 glucosides: daidzin, glycitin and genistin at *t*=0 and after 15 min of reaction at 30 °C. Error bars represent standard deviations (*n*=3).

The hydrolytic activity of *Ahe*GH1 towards three isoflavones glucosides (daidzin, glycitin, genistin) was assessed over 15 minutes incubation at 30 °C (Figure 6). At the control reaction, where no enzyme was added, no hydrolysis occurred, and the three isoflavone glucosides remained intact. When *Ahe*GH1 was added to the reaction, 100 % conversion to the correspondent aglycons, daidzein, glycitein and genistein was consistently achieved. It is important to highlight that this hydrolysis occurs in the presence of 30 % DMSO in addition to 15 % ethanol present in the reaction, as both solvents have been used to solubilise the isoflavone glucosides in their stock solutions. These results are in line with the findings from the activity and stability assays in the presence of different co‐solvents explained above.

### Performance of AheGH1 over soybean flour

**Figure 7:**
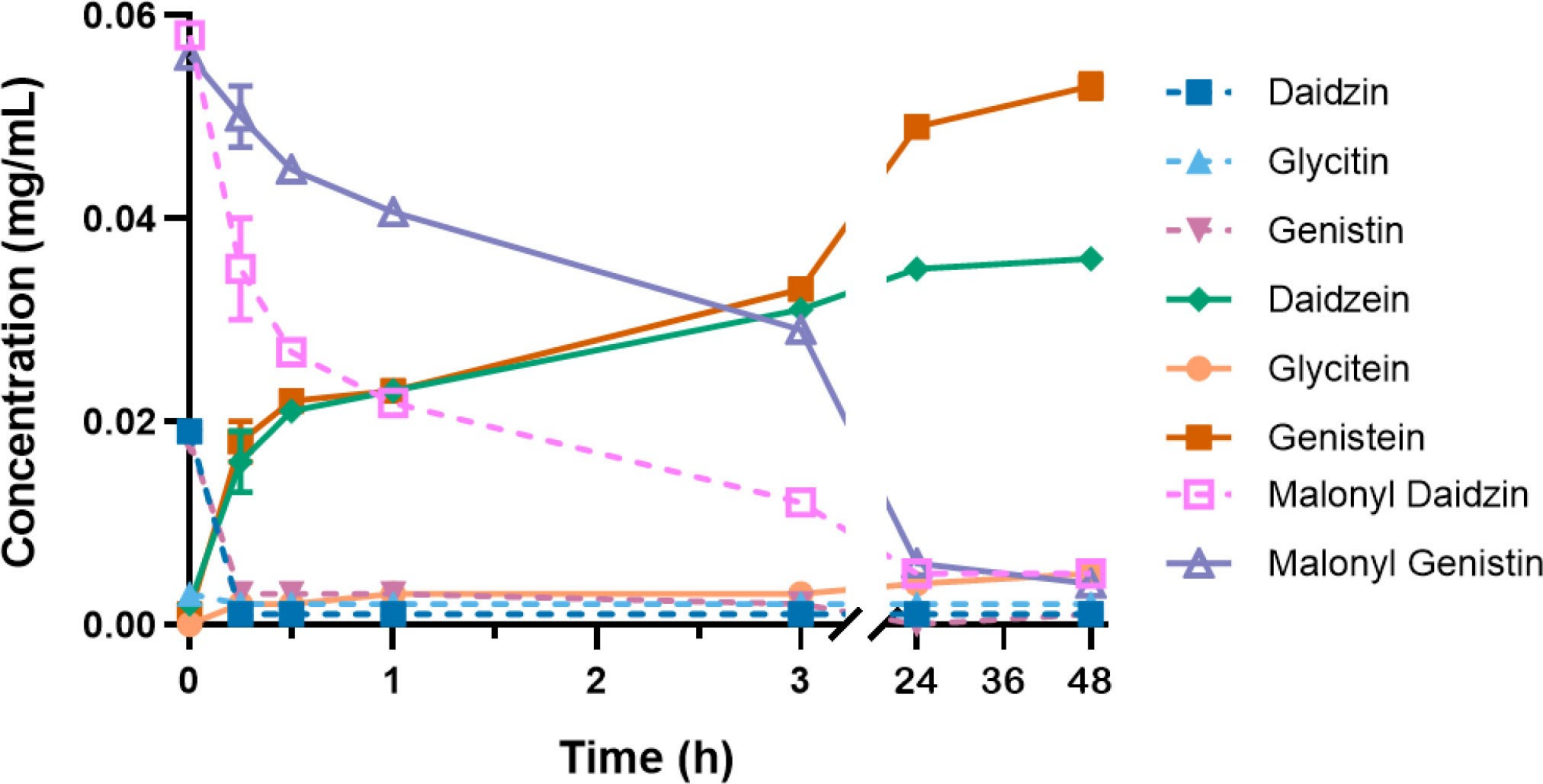
Hydrolysis reaction over soybean isoflavones glucosides at *t*=0 and after 15 min, 30 min, 1 h, 3 h, 24 h and 48 h at 30 °C when AheGH1 is present in the reaction. Error bars represent standard deviations (*n*=3).

Following the initial testing of *Ahe*GH1 with the synthetic isoflavone glucosides, the performance of the enzyme was tested with a real isoflavone mixture extracted from soybean flour. In this case, the reaction was followed over time to monitor the hydrolysis of the isoflavone glucosides and aliquots were taken from the biotransformation at time 0 and after 15 min, 30 min, 1 h, 3 h, 24 h and 48 h. The evolution in the hydrolysis rate achieved by *Ahe*GH1 is represented in Figure 7.

After 15 minutes of incubation, almost no isoflavone glucosides remained in the mixture, matching what had been observed with the pure standards. However, the amount of aglycons in the sample treated with *Ahe*GH1 continued to increase as the incubation time progresses. Concurrently, a decrease in two additional peaks was observed, which matched the standards malonyl daidzin and malonyl genistin (Figures S8–S13). The hydrolytic capacity of *Ahe*GH1 had initially only been evaluated towards the 7‐*O*‐β‐d‐glucosides daidzin, glycitin and genistin. After quantification, the increase in aglycons closely matched the decrease observed for the malonyl‐glucosides (Figure 7), confirming that the enzyme was also capable of hydrolysing this form, albeit at a slower rate.

## Conclusions

The extremozyme *Ahe*GH1 was successfully cloned, purified and characterised. Its performance was tested under different conditions usually found in industrial food processes such as glucose, fructose, different co‐solvents, broad range of temperatures and pH values showing promising results. The enzyme showed good tolerance to sugar (glucose and fructose) along with excellent performance in the presence of organic solvents, in particular ethanol. *Ahe*GH1 also exhibited wide pH stability and broad thermostability, making of it an outstanding candidate for its application in the food industry.

As a particular example, the evaluation of the hydrolytic capacity of *Ahe*GH1 towards the most common isoflavone glucosides found in soybeans, concluded that the enzyme can efficiently convert isoflavone glucosides into aglycones, and hence, it constitutes a promising candidate for the enzymatic production of soybean isoflavones.

## Experimental Section

**Chemicals and materials**: Commercially available reagents, organic solvents, and media were purchased from ACROS Organics, ThermoFisher Scientific, Merck, or Sigma‐Aldrich. The synthetic gene was purchased from Thermo Fisher Scientific and plasmid DNA purification kit from Macherey‐Nagel. DNA ladder, protein marker and restriction were purchased from New England Biolabs. Soybean flour and isoflavone standards were purchased from Merck and Carbosynth.

**Discovery of Ahe sequence**: A protein BLAST search was performed using the sequence of the halophilic β‐glucosidase *BglA* from *Halothermothrix orenii*[^43^, ^44^] against the *Alicyclobacillus* genera. *BglA* was used as model protein for its excellent activity and stability on a broad variety of conditions. *Alicyclobacillus* genera was selected because of its acidophilic properties. Candidate sequences from different *Alicyclobacillus* species were obtained, and *Ahe* was selected for showing better tolerance to acidic pH.

**Microbial strains and plasmids**: The synthetic gene coding for *A. herbarius* β‐GH1 (*Ahe*GH1), with restriction enzymes BamHI and HindIII flanking the sequences as restriction sites, was codon optimised for *E. coli* and then ordered from GeneArt (ThermoFisher) in pMA, a commercial cloning vector. The gene was digested with BamHI and HindIII and ligated into pCH93b, an expression vector produced in‐house,^[45]^ which includes a C‐terminal poly‐His tag for purification. *E. coli* strain XL10‐Gold harbouring the plasmid was grown at 37 °C in LB medium supplemented with ampicillin (0.1 mg mL^−1^). The gene was sequenced to confirm that the cloning was successful.

**Expression and purification**: Cells of *E. coli* BL21(DE3) harbouring the recombinant plasmid were grown at 37 °C in LB medium supplemented with ampicillin (0.1 mg mL^−1^). When the OD_600_ was between 0.6–0.8, isopropyl β‐d‐1‐thiogalactopyranoside (1 mM) was added to induce protein expression and the culture was left at 30 °C overnight. Cells were harvested at 4500 *g*, 4 °C, 20 min and the pellet stored at −20 °C until purification.

The cell pellet was resuspended in buffer (HEPES (50 mM), sodium chloride (150 mM), imidazole (10 mM), pH 7.5) and cells were broken by sonication (6 min cycle, 5 s on, 5 s off, 50 % amplitude, 1/4 inch probe, Fisherbrand™ Model 120 Sonic Dismembrator). The lysate was collected by centrifugation for 1 h at 14 500 *g* and 4 °C, and the pellet was discarded.

The supernatant was then filtered through PES 0.45 μm filters before loading it onto a IMAC column previously loaded with NiSO_4_ 0.1 M and washed with loading buffer (HEPES (50 mM), sodium chloride (150 mM), imidazole (10 mM), pH 7.5). The column was washed with loading buffer until a plateau in the UV_280_ absorbance was reached. Low affinity binding proteins were eluted using a step gradient 10 % elution buffer and the protein of interest was eluted using 100 % elution buffer (HEPES (50 mM), sodium chloride (150 mM), imidazole (300 mM), pH 7.5). The purified enzyme was dialysed overnight. Protein quantification was performed by measuring absorbance at 220, 250 and 280 nm using a BioTek Take3 Microplate reader using predicted extinction coefficients (54 854.15 Da, 108 415 M^−1^ cm^−1^).^[23]^

**Gel filtration**: Gel filtration chromatography was performed on a Superdex 200 10/300 GL column (GE Healthcare), using a mobile phase consisting of Tris⋅HCl (50 mM), KCl (100 mM), pH 7.5. Injection volume: 750 μL, flow rate 0.75 mL min^−1^. Samples were prepared to a final protein concentration of approximately 1 mg mL^−1^. A calibration curve was generated using the Sigma‐Aldrich Gel Filtration Markers Kit for Protein Molecular Weights 12 000–200 000 Da (MWGF200).

Thermal‐shift assay: 6 μL of *Ahe*GH1 (8.8 mg mL^−1^) was mixed with 9 μL SYPRO™ Orange Protein Stain (Sigma‐Aldrich) solution (prepared by diluting 0.7 μL in 250 μL water). Thermal‐shift assays were carried out using the MiniOpticon Real‐time thermocycler (Bio‐rad), over a temperature gradient of 15–99 °C, increasing at a rate of 2 °C min^−1^. The final melting temperature (*T*_m_) was calculated from the derivative of each sigmoidal, melting curve, as an average of quadruple values.

Crystallisation of AheGH1: *Ahe*GH1 crystals were grown using the sitting drop vapour diffusion technique, using an Orxy4 crystallisation robot (Douglas Instruments). Briefly, 400 nL drops comprising 10 mg mL^−1^ *Ahe*GH1, in 10 mM HEPES pH 7.5, 100 mM NaCl. Drops were set up in three‐drop, round CrystalQuick 96‐well plates (Greiner Bio‐One). Each reservoir contained 100 μL of 96 different crystallisation conditions from the PACT Premier screen (Molecular Dimensions). *Ahe*GH1 crystals grew after 5 days in condition F2 (0.2 M NaBr, 20 % (*w*/*v*) PEG 3350, 0.1 M Bis Tris Propane pH 6.5, at room temperature. Crystals were cryoprotected 0.1 M Bis Tris propane pH 6.5, 30 % (*w*/*v*) PEG 3350 and 30 % ethylene glycol cryocooled in liquid nitrogen.

**Data collection and 3D structure determination of AheGH1**: X‐ray diffraction data were collected on the I04 beamline at the Diamond Light Source (DLS Didcot, UK). Diffraction data were reduced using XDS^[46]^ and anisotropically truncated and scaled with STARANISO.^[47]^ Data collection statistics are reported in Table S1.

The structure was solved by molecular replacement using MOLREP^[48]^ from the CCP4 suite using the crystal structure of a thermostable β‐glucosidase from *H. orenii* as a search model (PDB ID: 4PTV.^[49]^ The model was further built using Coot^[50]^ and refined using phenix.refine^[51]^ and BUSTER.^[52]^ Water molecules were added using ARP/wARP suite^[53]^ and manually inspected in Coot. The final model was inspected and validated with MolProbity.^[54]^ Coordinates and structure factors of the *Ahe*GH1 have been deposited in the Protein Data Bank (www.rscb.org) with accession code 6YN7.

**Activity assay and kinetics**: Standard β‐glucosidase activity was determined spectrophotometrically using 10 mM *p*‐nitrophenyl‐β‐d‐glucopyranoside (*p*NPG) at 25 °C. 10 μL of a suitable enzyme dilution was added to a 96‐well plate per triplicate. Immediately before the assay, 0.29 mL of the reaction solution HEPES (50 mM), *p*NPG (10 mM), pH 7.4 were added and the *p*‐nitrophenol formation was followed at 420 nm for 10 min. The specific activity [U mg^−1^] was expressed as μmol of product formed per minute per milligram of protein. The extinction coefficient used for the *p*‐nitrophenol was calculated to be 8.64 mM^−1^cm^−1^.

To measure the kinetic properties of the studied enzyme, different concentrations of the substrate were used, and the enzymatic activity was measured using the same method as in the standard activity assay. Data were then plotted and fitted to a substrate inhibition curve^[31]^

$$\left(v=\frac{k_{cat}}{1+\frac{K_{m}}{\left[S\right]}+\frac{\left[S\right]}{K_{i}}}\right)$$

by using GraphPad Prism 8.

### Synthetic isoflavones standards

*Stock solutions*: Stock solutions for the β‐glucoside isoflavones (daidzin, glycitin and genistin) and for the aglycon isoflavones (daidzein, glycitein and genistein) were prepared from authentic samples (1 mg/mL in ethanol for daidzin, daidzein and genistein and 1 mg mL^−1^ in DMSO for genistin, glycitin and glycitein). Calibration solutions were prepared by dilution of the main stock solutions.

*Enzymatic reaction*: Prior to the testing of the enzyme in the real matrix, a solution containing the three main isoflavone glucosides present in soybeans (daidzin, genistin and glycitin) was prepared in triplicate. 100 μL sample containing the three isoflavones glucosides (150 μg mL^−1^ final concentration) and the enzyme (0.05 mg mL^−1^ final concentration) were left in agitation at 30 °C for 15 min. After that time, 450 μL of ACN were added to stop the enzymatic reaction and 450 μL of distilled water were added to top up until 1 mL total volume. The samples were then analysed by HPLC.

*Isoflavone extraction from soybean flour*: 1 g of soybean flour was weighed out and suspended in 4 mL of ethanol and 16 mL of distilled water. The sample was then sonicated for 20 min (5 s on, 5 s off, 60 % amplitude, 1/4 inch probe, Fisherbrand™ Model 120 Sonic Dismembrator) centrifuged for 30 min at 4500 *g* and filtered using a 0.45 μm filter.

*Enzymatic biotransformation*: After the extraction, 0.5 mg mL^−1^ of *Ahe*GH1 were added to a tube containing 3 mL of soybean extraction, in triplicate, and the samples were left in shaking at 30 °C. To follow the progress of the biotransformation, 100 μL aliquots were taken from the sample after 15 min, 30 min, 1 h, 3 h, 24 h and 48 h and transferred to HPLC vials. 450 μL of ACN and 450 μL of distilled water were added to stop the enzymatic reaction. The samples were then filtered (0.45 μm) and analysed by HPLC.

*Reversed‐phase HPLC analysis of conversions*: Samples were analysed using a ThermoFisher Ultimate 3000 Reverse‐phase HPLC (diode array detector) on a Waters XBridge C_18_ column (3.5 μm, 2.1×150 mm) with the following method: A: 0.1 % TFA in water, B: 0.1 % TFA in acetonitrile. Gradient: 0 min 95 % A 5 % B; 1 min 95 % A 5 % B; 5 min 5 % A 95 % B; 5.10 min 0 % A 100 % B; 6.60 min 0 % A 100 % B; 7 min 95 % A 5 % B; 10 min 95 % A 5 % B. Injection volume 2 μL, at 45 °C with a flow rate of 0.8 mL/min. Retention times in minutes: daidzin (3.17), glycitin (3.22), genistin (3.40), malonyl‐daidzin (3.44), malonyl‐genistin (3.62), daidzein (3.76), glycitein (3.82), genistein (4.04).

Conversions were calculated from a calibration curve of authentic standards. Peak areas were manually integrated, and the correlation coefficients were obtained using the Microsoft Excel® linear regression model application.

## Funding

L.D. was supported by the UK Engineering and Physical Sciences Research Council [grant no. EP/L015633/1]. C.H. was supported by the Biotechnology and Biological Sciences Research Council through the iCASE scheme in collaboration with Johnson Matthey [grant no. BB/M008770/1]. L.J.G. is grateful for funding from the Università degli Studi di Milano “Linea 2”.

## Conflict of interest

The authors declare no conflict of interest.

## Supporting information

As a service to our authors and readers, this journal provides supporting information supplied by the authors. Such materials are peer reviewed and may be re‐organized for online delivery, but are not copy‐edited or typeset. Technical support issues arising from supporting information (other than missing files) should be addressed to the authors.

Supplementary
