# Supplementary material for: Release of Soybean Isoflavones by Using a β‐Glucosidase from Alicyclobacillus herbarius
Source: Chembiochem. 2020 Dec 30;22(7):1223–31. doi: 10.1002/cbic.202000688 (PMC8048572; doi:10.1002/cbic.202000688)
Supplement: Supplementary file 1 — Supplementary [file CBIC-22-1223-s001.pdf]

ChemBioChem

Supporting Information

Release of Soybean Isoflavones by Using a β-Glucosidase from *Alicyclobacillus herbarius*

Lidia Delgado[+], Christian M. Heckmann[+], Flavio Di Pisa, Louise Gourlay, and Francesca Paradisi*

Supporting information

Table of contents

<u>Sequence alignment</u>

Sequence alignment based on the structural alignment of *Ahe*GH1 chain B (Ahe) and BglB (PDB: 2O9P), created using pymol.

<u>Supporting figures:</u>

Figure S1: SDS-PAGE gel of a typical purification of *Ahe*GH1. cfe: cell-free extract, FT: flow-through (IMAC purification), wash: eluted with 10% elution buffer, pure: eluted with 100% elution buffer.

Figure S2: Graphic showing the correlation between the log10 (MW) and the ratio Ve/Vo. The protein standards are represented in blue while *Ahe*GH1 is represented in green

Figure S3: Overview of sequence and structure conservation of AheGH1with other glucosidases.

Figure S4: Structural superposition of the active sites of *Ahe*GH1 (ice blue) and the homologous proteins *BglA* (yellow, pdb code 1E4I) and *BglB* (pink, pdb code 2O9P)

Figure S5: Effect of the incubation in a different range of pH on AheGH1 stability. The activity was tested using the standard activity assay at 25 ºC. Activity expressed relative to the activity at t=0. Error bars represent standard deviations (n = 3).

Figure S6: Effect of the incubation in a different range of temperatures on AacGH1 stability. The activity was tested using the standard activity assay at 25 ºC. Activity expressed relative to the activity at t=0. Error bars represent standard deviations (n = 3).

Figure S7: Thermofluorometric melting profile of *Ahe*GH1, over a temperature gradient of 15-99 °C, increasing at a rate of 2 °C/min.

Figure S8: Chromatogram of soy flour extract with and without enzymatic treatment (48 h), 260 nm. 1: daidzin, 2: glycitin, 3: genistin, 4: malonyl-daidzin, 5: malonyl-genistin, 6: daidzein, 7: glycitein, 8: genistein.

Figure S9: Chromatogram of commercial isoflavone standards, 260 nm.

Figure S10: UV spectra of daidzin (top) and glycitin (bottom), obtained with the diode array detector. Left: authentic standard; right: corresponding peak in soy-flour-extract chromatogram. Black: spectrum at peak, green: spectrum at 50% peak height leading the peak, pink: spectrum at 50% peak height tailing the peak.

Figure S11. UV spectra of genistin (top) and malonyl-daidzin (bottom), obtained with the diode array detector. Left: authentic standard; right: corresponding peak in soy-flour-extract chromatogram. Black: spectrum at peak, green: spectrum at 50% peak height leading the peak, pink: spectrum at 50% peak height tailing the peak. The peaks for genistin and malonyl-daidzin are not well separated in the soy-flour extract.

Figure S12. UV spectra of malonyl-genistin (top) and daidzein (bottom), obtained with the diode array detector. Left: authentic standard; right: corresponding peak in soy-flour-extract chromatogram. Black: spectrum at peak, green: spectrum at 50% peak height leading the peak, pink: spectrum at 50% peak height tailing the peak.

Figure S13. UV spectra of glycitein (top) and genistein (bottom), obtained with the diode array detector. Left: authentic standard; right: corresponding peak in soy-flour-extract chromatogram. Black: spectrum at peak, green: spectrum at 50% peak height leading the peak, pink: spectrum at 50% peak height tailing the peak. The peaks for daidzin and glycitein are not well separated in the soy-flour extract.

Supporting tables:

Table S0: Table for size exclusion chromatography in the conditions referred. Ve makes reference to the elution volume expressed in minutes. MW is the molecular weight of each standard in kDa. Ve/Vo is the elution volume divided by the void volume of the column.

Table S2: Data collection and refinement statistics. Data collection and refinement statistics for X-ray diffraction data collected on a single crystal of *Ahe*GH1. Values in parenthesis correspond to the high-resolution shell. For cross-validation, 5% experimental reflections were randomly selected to calculate the R_{free}.

Sequence alignment

Sequence alignment based on the structural alignment of *Ahe*GH1 chain B (Ahe) and *BglB* (PDB: 2O9P), created using pymol.

```
Ahe          TREFISFPQDFLFGTATASYQIEGAVHEDGRGESIWDRFSHTPGKVYQGHTGDVACDHYHRYR
2O9P         NTFI--FPATFMWGTSTSSYQIEGGTDEGGRTPSIWDTFCQIPGKVIGGDCGDVACDHFHHFK
                   **..*..**.*.******...*.**..****.*.     *..*..*******.*...

Ahe          EDVALMKELGIPAYRFSIAWPRIFPEKGMKNEAGLDFYRRLLEALHEADIRSFVTLYHWDLPQ
2O9P         EDVQLMKQLGFLHYRFSVAWPRIMPAAGIINEEGLLFYEHLLDEIELAGLIPMLTLYHWDLPQ
             ***.***.**...****.*****.*..*..**.**.**..**.....*......*********

Ahe          WLQDRGGWANRDTAEYFAEYASLIYERLGDGIDAFITHNEPWCAAFLGHGFGVHAPGHTDWRE
2O9P         WIEDEGGWTQRETIQHFKTYASVIMDRFGERINWWNTINEPYCASILGYGTGEHAPGHENWRE
             *..*.***..*.*. .* ***.*..*.*  *....*.***.**..**.*.*.*****..***

Ahe          AFQAAHHILYSHGLAVQAHRASSHKGQIGITLNFTWVDAATDSATDQAAAEVSHAFNNRWFLE
2O9P         AFTAAHHILMCHGIASNLHKEKGLTGKIGITLNMEHVDAASERPEDVAAAIRRDGFINRWFAE
             **.******..**.*...*..   .*.******...****.....*.***.....*.****.*

Ahe          PVAGRGYPQEFQQLVEQRIGQFDFVRQGDLAVIAEPIDFLGINFYTRSVVAANPDDALFGLRT
2O9P         PLFNGKYPEDMVEWYGTYLNGLDFVQPGDMELIQQPGDFLGINYYTRSIIRSTNDASLLQVEQ
             *..  .**........    .***..**...*.  .******.****......   *.....

Ahe          LEA--NRTEMGWEIHPDSLYRLLTWVQSVTGQ-LPLYITENGAAFADEPVNGRVEDVRRIHYI
2O9P         VHMEEPVTDMGWEIHPESFYKLLTRIEKDFSKGLPILITENGAAMRDELVNGQIEDTGRQRYI
                  ..*.*******.*.*.***....    **..*******..**.***..**..*..**

Ahe          ADHLEAAKRFVDAGGPLKGYFLWSFMDNFEWALGYSKRFGMVYVDYESQQRLVKDSGRWFSEQ
2O9P         EEHLKACHRFIEEGGQLKGYFVWSFLDNFEWAWGYSKRFGIVHINYETQERTPKQSALWFKQM
             ..**.*..**...**.*****.***.******.*******.*...**.*.*..*.*..**...

Ahe          IAAHKGQVR
2O9P         MAKNGF---
             .*
```

Supporting Figures

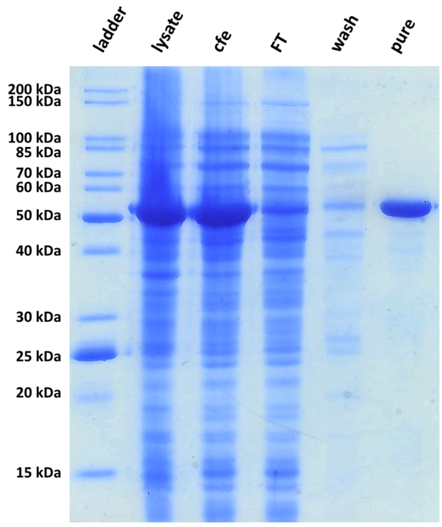

Figure S1: SDS-PAGE gel of a typical purification of *Ahe*GH1. cfe: cell-free extract, FT: flow-through (IMAC purification), wash: eluted with 10% elution buffer, pure: eluted with 100% elution buffer.

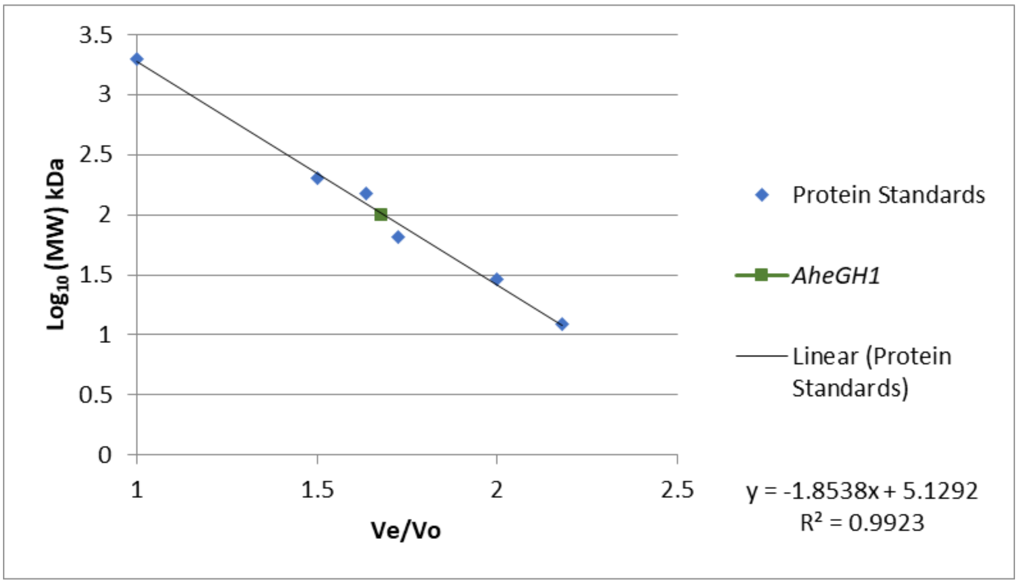

Figure S2: Graphic showing the correlation between the log10 (MW) and the ratio Ve/Vo. The protein standards are represented in blue while *Ahe*GH1 is represented in green

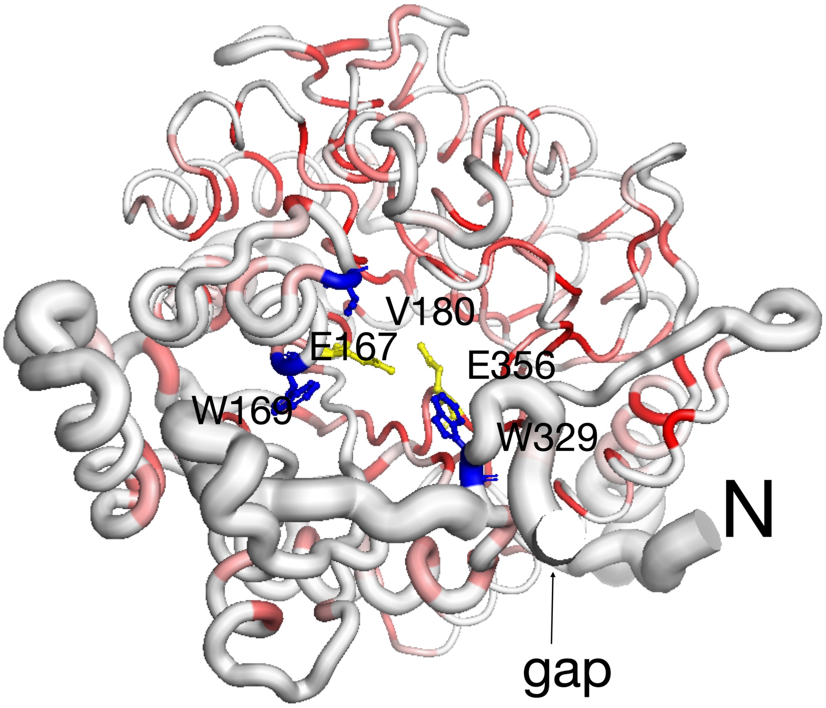

Figure S3: Overview of sequence and structure conservation of *Ahe*GH1 with other glucosidases. Sausage representation indicating sequence and structure conservation of *Ahe*GH1 with all other glycosyl hydrolases deposited in the Protein Data Bank was calculated using the ENDscript 2 server (http://endscript.ibcp.fr/ESPript/ENDscript/), using default parameters. Red shading indicates sequence conservation, with the darker colouring representing the highest sequence conservation. Structure conservation is indicated by sausage thickness with the least conserved regions indicated by increased thickness. The catalytic glutamate residues are indicated, as are the conserved residues shared in the loops of *BglA* and *BglB* that form the entrance to the active site tunnel. The gap in the *Ahe*GH1, due to lack of electron density is indicated.

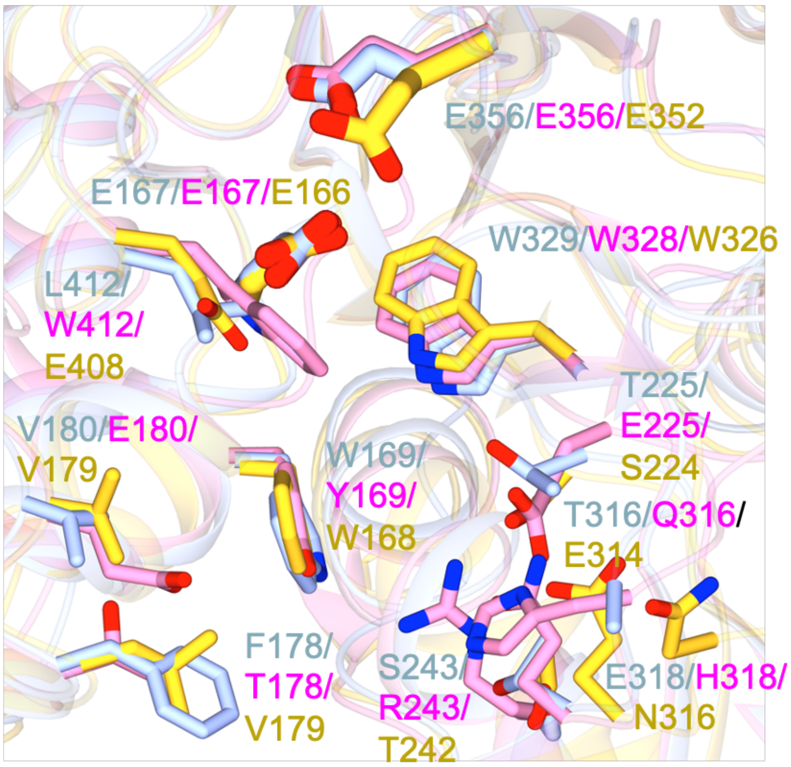

Figure S4: Structural superposition of the active sites of *Ahe*GH1 (ice blue) and the homologous proteins *BglA* (yellow, pdb code 1E4I) and *BglB* (pink, pdb code 2O9P). The residues delineating the pocket are shown as sticks, labeled and colored accordingly. The catalytic glutamate residues are depicted with thicker sticks. The *Ahe*GH1-E318 side-chain is missing. This figure was generated using CCP4mg.

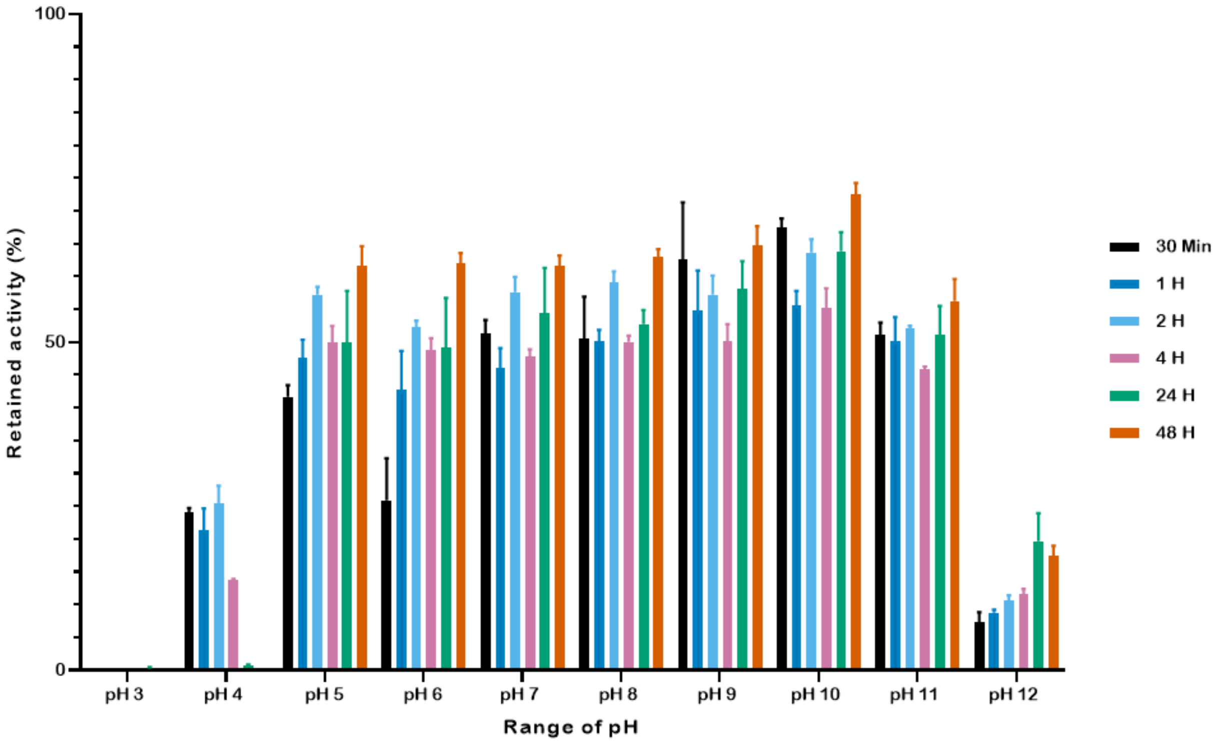

Figure S5: Effect of the incubation in a different range of pH on *Ahe*GH1 stability. The activity was tested using the standard activity assay at 25 ºC. Activity expressed relative to the activity at t=0. Error bars represent standard deviations (n = 3).

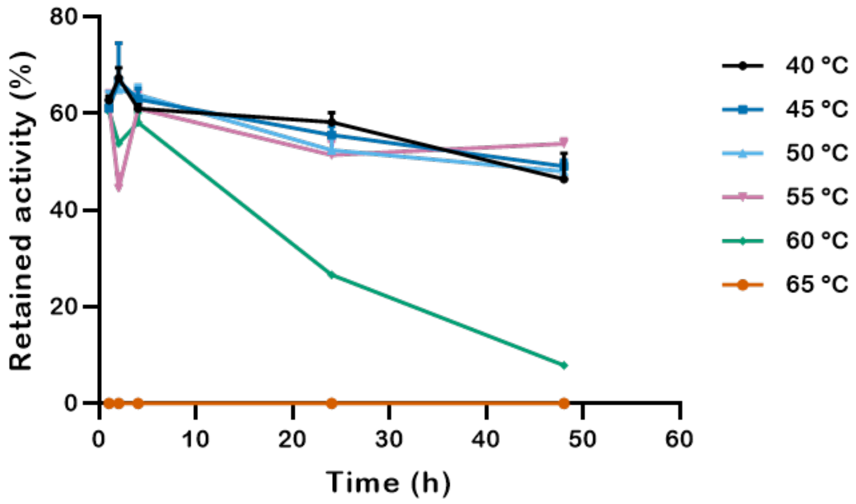

Figure S6: Effect of the incubation in a different range of temperatures on *Aac*GH1 stability. The activity was tested using the standard activity assay at 25 ºC. Activity expressed relative to the activity at t=0. Error bars represent standard deviations (n = 3).

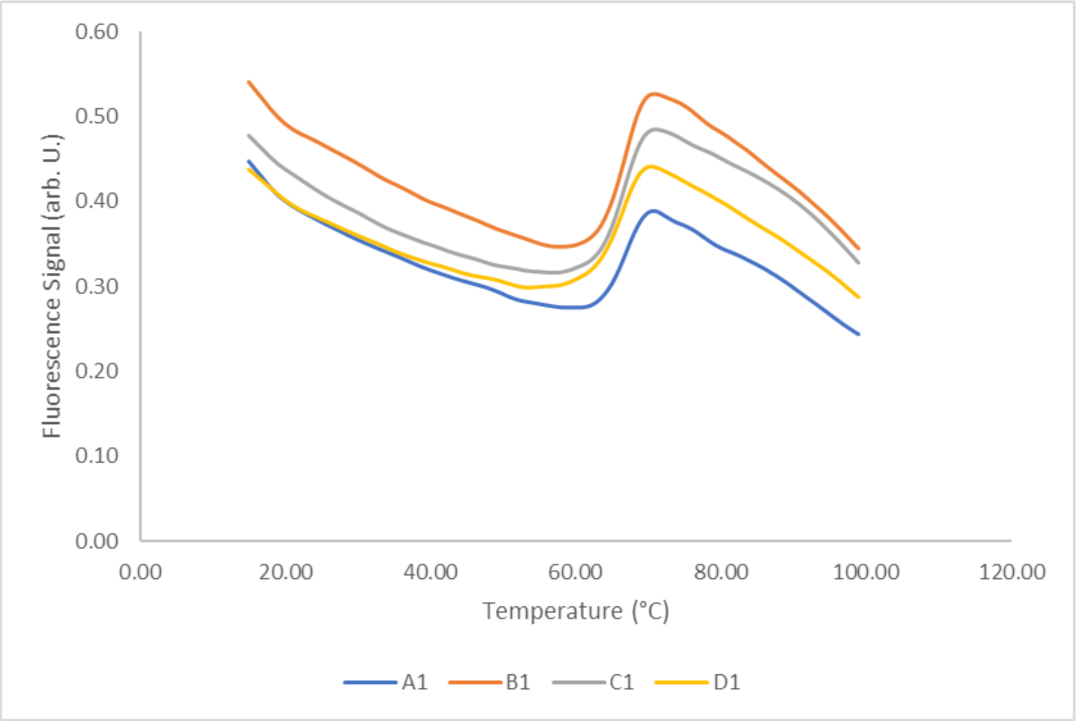

Figure S7: Thermofluorometric melting profile of *Ahe*GH1, over a temperature gradient of 15-99 °C, increasing at a rate of 2 °C/min.

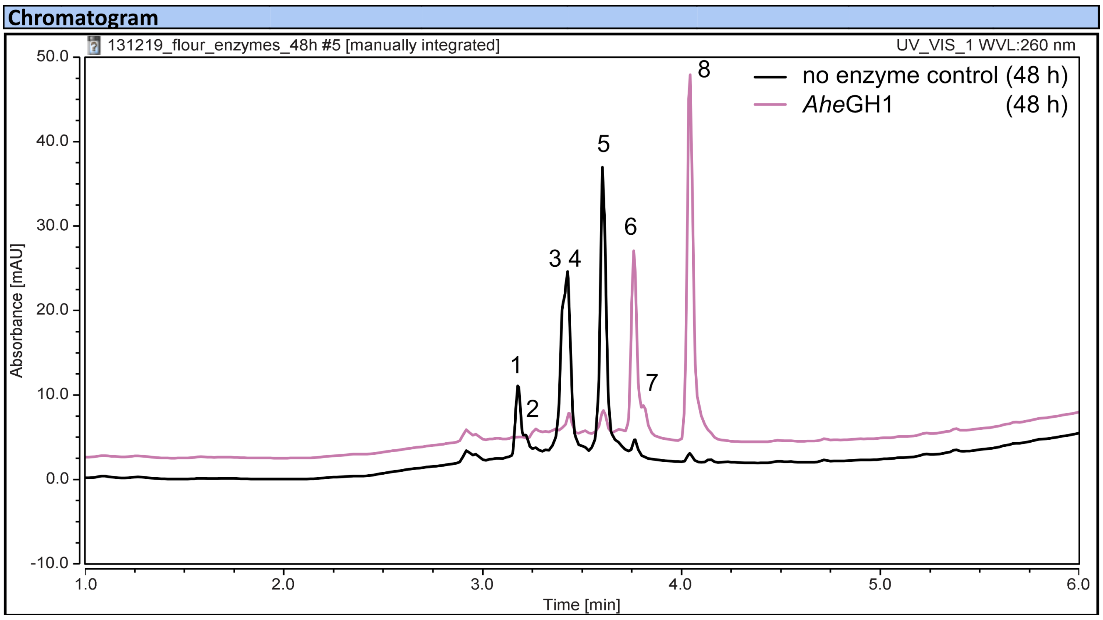

Figure S8: Chromatogram of soy flour extract with and without enzymatic treatment (48 h), 260 nm. 1: daidzin, 2: glycitin, 3: genistin, 4: malonyl-daidzin, 5: malonyl-genistin, 6: daidzein, 7: glycitein, 8: genistein.

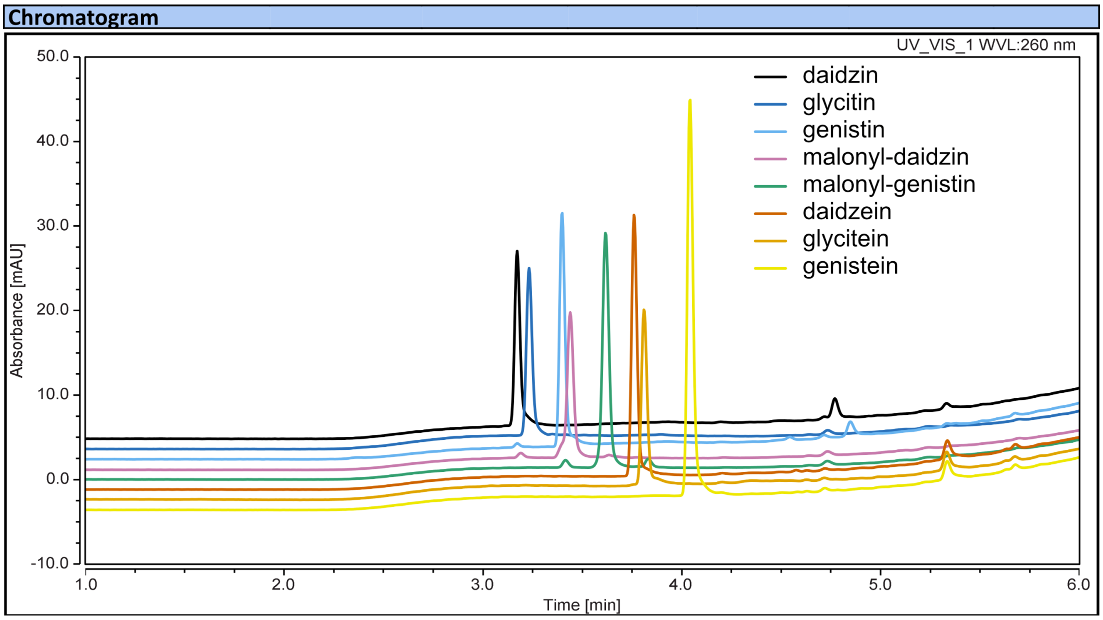

Figure S9: Chromatogram of commercial isoflavone standards, 260 nm.

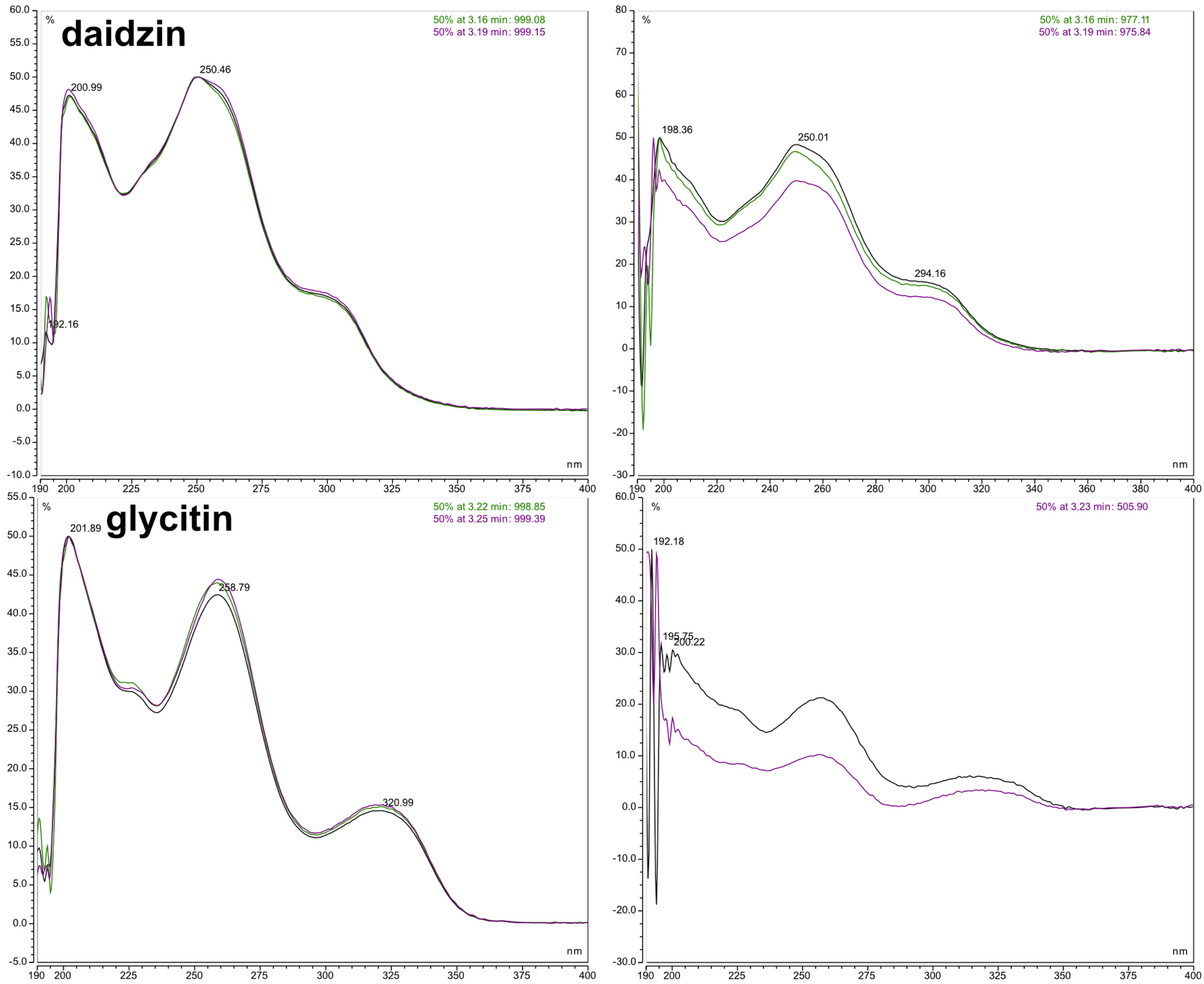

Figure S10: UV spectra of daidzin (top) and glycitin (bottom), obtained with the diode array detector. Left: authentic standard; right: corresponding peak in soy-flour-extract chromatogram. Black: spectrum at peak, green: spectrum at 50% peak height leading the peak, pink: spectrum at 50% peak height tailing the peak.

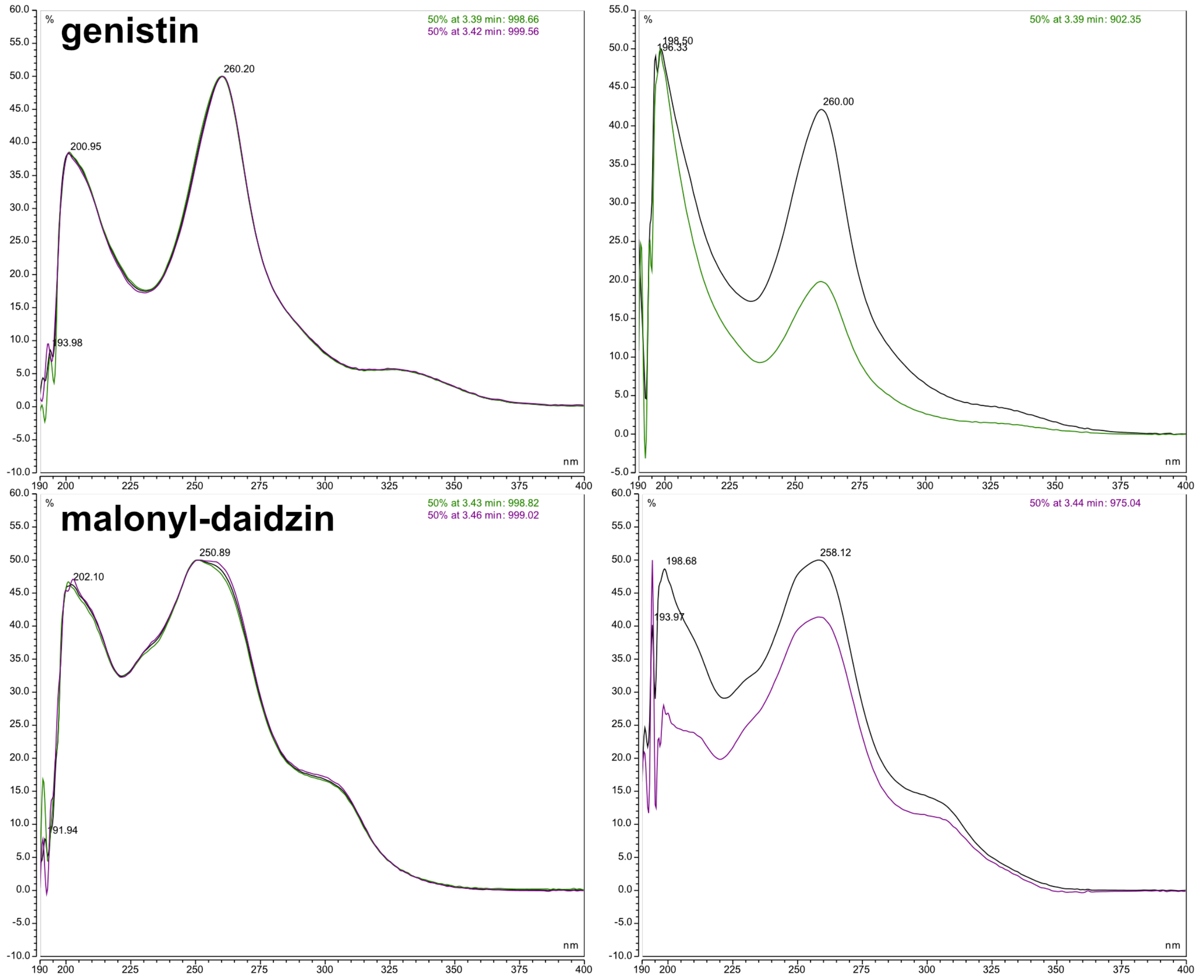

Figure S11. UV spectra of genistin (top) and malonyl-daidzin (bottom), obtained with the diode array detector. Left: authentic standard; right: corresponding peak in soy-flour-extract chromatogram. Black: spectrum at peak, green: spectrum at 50% peak height leading the peak, pink: spectrum at 50% peak height tailing the peak. The peaks for genistin and malonyl-daidzin are not well separated in the soy-flour extract.

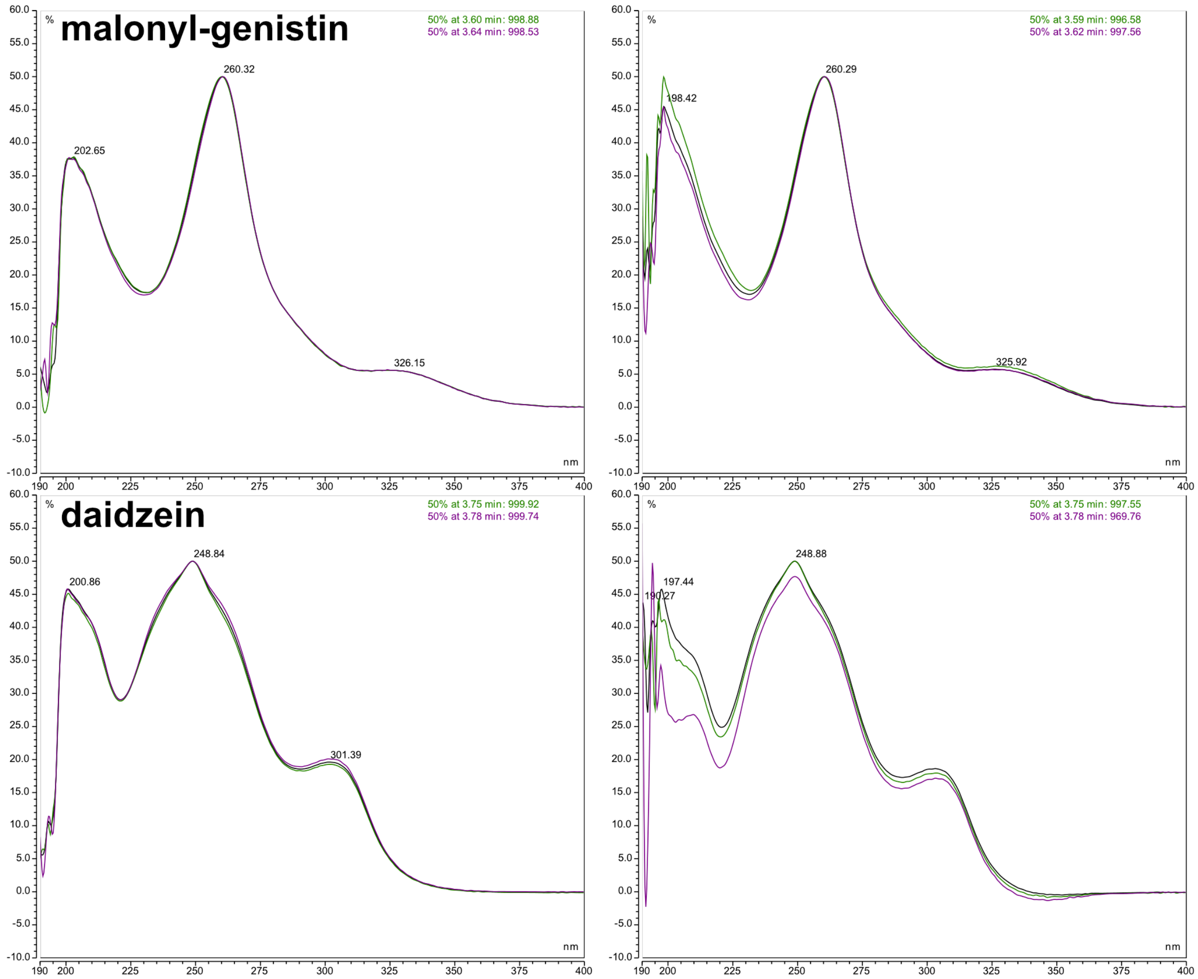

Figure S12. UV spectra of malonyl-genistin (top) and daidzein (bottom), obtained with the diode array detector. Left: authentic standard; right: corresponding peak in soy-flour-extract chromatogram. Black: spectrum at peak, green: spectrum at 50% peak height leading the peak, pink: spectrum at 50% peak height tailing the peak.

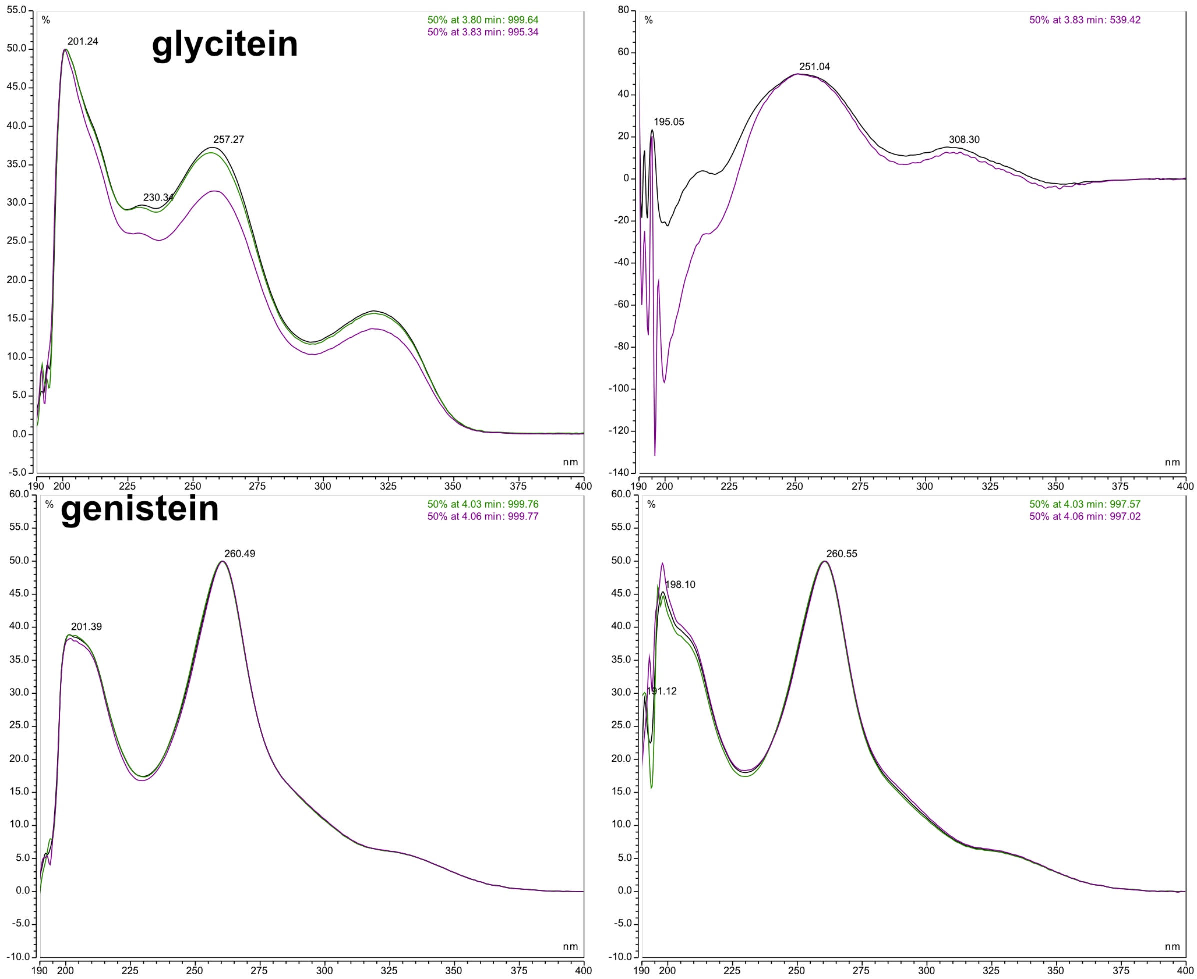

Figure S13. UV spectra of glycitein (top) and genistein (bottom), obtained with the diode array detector. Left: authentic standard; right: corresponding peak in soy-flour-extract chromatogram. Black: spectrum at peak, green: spectrum at 50% peak height leading the peak, pink: spectrum at 50% peak height tailing the peak. The peaks for daidzin and glycitein are not well separated in the soy-flour extract.

Supporting Tables

Table S1: Table for size exclusion chromatography in the conditions referred. Ve makes reference to the elution volume expressed in minutes. MW is the molecular weight of each standard in kDa. Ve/Vo is the elution volume divided by the void volume of the column.

Sample	KDa	min	Volume (mL)	Ve/Vo	Protein Standards log 10 (MW)	Estimated Kda
Blue dextran	2000	11	8.25	1.00	3.30	
B-amylase	200	16.5	12.38	1.50	2.30	
Cytochrome C	12.4	24	18.00	2.18	1.09	
Carbonic anhidrase	29	22	16.50	2.00	1.46	
ADH	150	18	13.50	1.64	2.18	
Albumin	66	19	14.25	1.73	1.82	
Ahe GH1			13.87	1.68	2.00	99.75

Table S2: Data collection and refinement statistics. Data collection and refinement statistics for X-ray diffraction data collected on a single crystal of *Ahe*GH1. Values in parenthesis correspond to the high-resolution shell. For cross-validation, 5% experimental reflections were randomly selected to calculate the R_{free}.

	AHE (PDB code 6YN7)
**Crystal**	
Space group	P 1 2_1 1
Unit cell dimensions *a*, *b*, *c* (Å); β (°)	100.45 93.35 106.38; 98.70
**Data collection**	
Beamline	DLS I04
Wavelength (Å)	0.979
Resolution (Å)	105.16-1.98 (2.27-1.98)
Total reflections	401061 (16134)
Unique reflections	58817 (2941)
R_{merge}	0.17 (1.05)
$^{\#}R_{meas,}$	0.18 (1.15)
$I/\sigma(I)$	7.0 (1.5)
$^{+}CC_{1/2}$	0.996 (0.693)
Completeness (%) ellipsoidal	93.5 (66.5)
Redundancy	6.8 (5.5)
Wilson B-factor (Å)	22.70
**Refinement**	
Resolution (Å)	1.98
No. reflections	58600
R_{work} / R_{free}	24.7/29.8
No. atoms	
Protein	14243
Water	273
B factors	
Protein	26.7
Water	20.8
R.m.s. deviations	
Bond lengths (Å)	0.002
Bond angles (°)	0.456
Clash scores	5.44
Ramachandran	
Favored (%)	96.1
Allowed (%)	3.9

† Redundancy-independent merging *R* factor R_{meas} estimated by multiplying the conventional R_{merge} value by the factor $[N/(N-1)]^{1/2}$, where *N* is the data multiplicity.

+ $CC_{1/2}$ is the correlation coefficient of the mean intensities between two random half-sets of data.
